# Research and Medical Students: Some Notable Contributions Made in History

**DOI:** 10.31729/jnma.5078

**Published:** 2021-01-31

**Authors:** Pravakar Dawadi, Sabina Khadka

**Affiliations:** 1 Nepalese Army Institute of Health Sciences, Sanobharyang, Kathmandu, Nepal

**Keywords:** *evidence based practice*, *medical students*, *research*

## Abstract

Upon the commencement of the practice of modern medicine, the establishment of evidence based practice has played a crucial role in its advancement. Whether it be an expert medical practitioner or some beginner medical student who is in the early phase of pursuing their career, each of them can contribute in our own way. This article highlights some of the many contributions made in modern medicine by those early buds in the past.

The practice of medicine has started since the existence of humankind on the earth. But we cannot trace back the commencement of scientific and evidence-based medicine so far. The insight we are getting from current medical science is the output of people's laborious effort and contributions before us. The statement stating ‘we learn from our past’ is perfectly resonant with the practice of clinical medicine.

Hippocrates, who is commonly known to be the ‘Father of Modern Medicine,’ initiated the rational and logical medicine practice based on evidence. He formed a diagnostic system by integrating knowledge from the past based on clinical observation and logical reasoning. The rejection of the long-standing concept that some divine force caused illness was a revolutionary beginning of the modern medicine era. Hippocrates instead attributed the diseases to natural cause and insisted treatment should be based on observation, reasoning, and past experiences.^1^

Those past experiences or findings which are scientifically cataloged are crucial in the advancement of modern medicine. The countless researches conducted in various aspects of medical science prove to be strong evidence of our past achievements. Many people's contribution to such research activities in the medical field has taken us this far in modern medicine. In the pool of such people, medical students who are in the early phase of pursuing their career in medicine have also contributed a lot to medical research time and again.^2^

## HEPARIN

Heparin, a natural anticoagulant, has revolutionized the management and treatment of thromboembolic disorders and surgical procedures. Its discovery can be attributed to Jay Mclean (1890-1957), who identified a phosphatide anticoagulant in canine liver tissue while he was a second-year medical student at Johns Hopkins University in 1916.^3^ After the introduction of heparin in the 1940s in clinical practice, it has had a significant impact on the clinical course of surgery.

## RAYNAUD'S DISEASE/PHENOMENON

Raynaud's disease is a common vascular phenomenon that is recognized as a manifestation of a wide spectrum of clinical disorders.^4^ Its discovery can be traced back as early as 1862 by Auguste-Maurice Raynaud (1834-1881). In his medical doctorate thesis entitled Local Asphyxia and Symmetrical Gangrene of the Extremities, he described this phenomenon.^5^ Even after he completed his medical school, he continuously worked further expanding and updating his observations for the next 30 years.^6^

## ISLETS OF LANGERHANS

The study of the pancreas had begun in the 16^th^ century, but only secretory acini and ductal system were known so far.^7^ Paul Langerhans (1847-1888) studied medicine in Berlin. During his time in medical school, he made two important discoveries. He described the dendritic cells in the skin, which is now commonly known as Langerhans cells. Another discovery was the pancreatic islets. Langerhans resumed his research on the pancreas’ microscopic anatomy in 1868, which he started briefly in 1867. They completed in 6 months.^8,9^ Langerhans identified small clusters of ‘irregularly polygonal’ cells with clear cytoplasm scattered throughout the gland using pancreatic tissue of salamander, rabbit, and human.^10,11^ Twenty four years later, French histopathologist Edouard Laguesse (1861-1927) identified the islets of Langerhans’ as the site of internal secretion of the pancreas and later named it insulin.

## INSULIN

Charles Herbert Best (1899-1978) and Frederick Grant Banting (1891-1941) discovered insulin in 1921. After his study in the liberal arts program at the University of Toronto was interrupted due to World War I, Best switched course to physiology and biochemistry for preparation of medical degree after 1919. Banting was a 28 years old medical practitioner. John JR Macleod, a physiology teacher of Best, introduced him to Banting. They used Macleod's laboratory for their research project.

They were convinced that the extract of islets of Langerhans from a dog was crucial in preventing diabetes mellitus. They tested the pancreatic extract in a diabetic dog in 1921 and confirmed its efficacy in treating diabetes. Later, professor Macleod hired a biochemist, James Collip, to help purify the extract's active component. They were also successful in treating a diabetic patient with the extract.^12^

The patient was a fourteen-year-old boy Leonard Thompson expected to live only a few more weeks. But, after they treated the boy with the extract, he lived for another 13 years. He died of complications from a road accident. In 1923, Banting and Macleod were awarded the Nobel Prize in physiology and medicine for the discovery of insulin. They later shared the award with Best and Collip, respectively.^13^

## SINOATRIAL NODE

Martin William Flack (1882-1931), a medical student, was spending a holiday with his lecturer by studying the hearts of moles, mice, and frogs. His anatomy lecturer, Dr. Arthur Keith (1866-1955), returned to the holiday cottage from a cycle ride and was amused by his student's finding. Flack showed him the structures he discovered in the right auricle of the heart of a mole.^14^

Upon seeing the structure, Keith suspected it might be the atrioventricular node. It was identified by a Japanese anatomist, Sunao Tawara, a year before, which was confirmed by Flack and Keith themselves in an earlier study.^15^ Then they studied the heart of other mammals as well. It allowed them to establish the presence of a ‘sinoatrial node’ in other mammals too. While doing so, they also found the origin of the ‘dominating rhythm of the heart.^16^

Upon graduating in 1908, Flack further researched the rejuvenating effects of oxygen in athletes as a physiology lecturer in London Hospital.^17^ He was appointed as the first Director of the Medical Research Council for the Royal Air Force in 1919. There he continued to investigate cardiopulmonary physiology and established techniques for assessing the physical fitness of pilot candidates.

## KLUMPKE PARALYSIS

Augusta Klumpke (1859-1927) was initially destined to become a teacher until she read an article in a fashion magazine regarding a recently graduated woman in medicine in Paris. After her family moved to Paris in 1877, she admitted herself to the Faculty of Medicine.^18^ As she was fluent in French, German, and English, she did not have any hard time understanding contemporary literature.

Meanwhile, she diagnosed a brachial plexus palsy associated with Horner's syndrome while working at the Hôtel-Dieu. She presented the lesions of the inferior roots of the brachial plexus as her thesis.^19^ She won an Academy of Medicine prize for the research. Due to the male medical hierarchy, the award was not enough to get a hold of the internship opportunity. After repeated failed attempts and continuous lobbying, she was allowed to compete for an internship in 1886. She then became the first-ever female full intern in a Persian hospital.

## SPHINCTER OF ODDI

Guggero Oddi (1864-1913) was a fourth-year medical student at the University of Perugia. He studied the actions of the sphincter present at the distal end of the common bile duct.^20,21^ He established the relation between the sphincter and the bile's controlled flow from the liver to the duodenum. He also recommended that sphincter dysfunction might be related to biliary tract disease. He shifted to the University of Bologna from the University of Perugia as it could not award him a medical degree. At Bologna, he studied the pressure changes across the sphincter.^22^

## PENICILLIN

Ernest Duchesne (1874-1912) was a 23-year-old medical student at l'École du Service de Santé Militaire in Lyon, France. His graduation thesis, ‘Contribution to the study of vital competition between microorganisms: antagonism between molds and microbes,’ demonstrated the capability of the fungus Penicillium glaucum to treat harmful bacterial infections caused by Escherichia coli or Salmonella typhi.^23^

He did in vivo experiments using guinea pigs. Duchesne proposed the action could be due to a toxin (antibiotic) released by the fungus. He also emphasized the therapeutic potential of the fungus. But, since Duchesne could not continue his research, his work was led to completion by Chain, Florey, and Jennings in 1942.

## ANESTHESIA

A medical student named William E. Clarke was the first to use ether anesthesia for surgery, as Lyman claimed.^24^ This event happened in Rochester, New York, in January 1842, inside a dental clinic. It enabled the dentist to extract a tooth painlessly. But the recognition for the use of ether anesthesia is often given to others, including Crawford Williamson Long (1815-1878). He was a young doctor in Georgia. In March 1842, he used ether upon a young man to excise a small cyst from his neck painlessly.^25^

## SPERMATOZOA

Johan Ham (1651-1723) was a medical student from Leiden who discovered spermatozoa for the first time in 1677. He took a specimen containing the urethral discharge of a man suffering from gonorrhea to Lewenhoeck (1632-1723). In the specimen, Ham found out tiny living ‘animalcules’ with tails.^26^ Leeuwenhoek, although being poorly educated, because of his passion for making lenses and studying biological tissues along with microorganisms made himself the father of microscopy.^27,28^

Ham was often credited for the identification of spermatozoa in the semen of rooster.^26^ He also pointed out the absence of spermatozoa in the semen of sterile men.^26^ He also proposed that those spermatozoa cannot survive beyond 24 hours.^29^ Lewenhoeck studied his own semen obtained by conjugal coitus and verified the presence of a small motile animalcule. A month later, Lewenhoeck published these findings in a letter to the Royal Society in London where he credited the discovery to Johan Ham.^29,30^

## CONCLUSIONS

These are a few notable achievements of medical science made by people while still in medical school. Lots of contributions have been made by the medical fraternity for the advancement and better understanding of modern medicine. We can now understand that students’ responsibility in medical school does not only start after they graduate. Instead, we can hope to contribute the medical science from the day we have begun our medical classes.

Whether it be a small task like assisting our professors in their fieldwork or be it some big task working on some ongoing research project, we would be contributing to the advancement of modern medicine while still studying for the same. These examples from our history keep us motivated and consistent on our path to greatness.
